# Identification and Characterization of Circular RNA as a Novel Regulator and Biomarker in Preterm Birth

**DOI:** 10.3389/fbioe.2020.566984

**Published:** 2020-12-02

**Authors:** Yuxin Ran, Nanlin Yin, Dongni Huang, Yangyu Zhao, Jing Yang, Hanwen Zhang, Hongbo Qi

**Affiliations:** ^1^Department of Obstetrics, The First Affiliated Hospital of Chongqing Medical University, Chongqing, China; ^2^Chongqing Key Laboratory of Maternal and Fetal Medicine, Chongqing Medical University, Chongqing, China; ^3^Joint International Research Laboratory of Reproduction and Development of Chinese Ministry of Education, Chongqing Medical University, Chongqing, China; ^4^Center for Reproductive Medicine, The First Affiliated Hospital of Chongqing Medical University, Chongqing, China; ^5^Department of Obstetrics & Gynecology, Peking University Third Hospital, Beijing, China

**Keywords:** preterm birth, circRNA, ceRNA network, mechanism, prediction

## Abstract

Preterm birth (PTB), as the leading cause of neonatal death, is a severe threat to maternal–fetal health. The diagnosis and treatment of PTB are difficult as its underlying mechanism still unknown. Circular RNA (circRNA) is an emerging molecule that plays an essential role in the pathological processes of various diseases. However, it is still unclear whether circRNAs are abnormal or involves in the PTB pathology. In this study, we analyzed RNA-seq data of peripheral blood from preterm and term pregnant women and verified with microarray data. There were 211 circRNA expression disorders in PTB, of which 68 increased and 143 decreased. Bioinformatics analysis revealed that the top 20 circRNAs competitively bind 68 miRNAs, thereby regulating 622 mRNAs mainly related to immunity, inflammation, and nerve activity, which may ultimately contribute to the occurrence of PTB. Moreover, 6 regulatory pairs, including hsa-MORC3_0001–hsa-miR-1248–CHRM2 were the core parts of this mechanism network, which might be therapeutic targets for PTB. Besides, ROC analysis indicated that hsa-ANKFY1_0025, hsa-FAM13B_0019, and hsa-NUSAP1_0010 (AUC = 0.7138, 0.9589, 1.000) have an excellent discrimination ability for PTB. Taken together, we explored for the first time the circRNA expression profile of PTB, and preliminarily analyzed its regulatory mechanism and predictive value for PTB, thus bringing new light to the diagnosis and treatment of PTB.

## Introduction

Preterm birth (PTB) is defined as a birth that occurs before 37 completed weeks of gestation (The Lancet, 2016). The average incidence of PTB globally is approximately 10.6%, while in China, it is 7.8% (Chawanpaiboon et al., 2019). An estimated 14.8 million premature babies were born across the globe annually, with the death rate as high as 17.8% (Liu et al., 2016). Survived premature babies are at higher risk than term babies of various health problems, among which the worst one is cerebral palsy (Demesi Drljan et al., 2016; Natarajan and Shankaran, 2016). Although PTB has become a terrible threat to global health, its specific molecular mechanism remains unclear. It is currently accepted that PTB is a multifactorial syndrome. Several risk factors including metabolic disorders, stress, aging, physical stimulation can promote PTB. Besides, immune/inflammation might be the most essential one (Romero et al., 2014). This situation largely limits the diagnosis and therapy of PTB (Muglia and Katz, 2010). Even the most widely used clinical test, fetal fibronectin (fFN), can only roughly assess the risk of PTB, but cannot suggest potential pathological changes before the occurrence of PTB (Berghella and Saccone, 2019). It would facilitate treatment and significantly improve the outcome of premature babies if PTB can be predicted earlier (Suff et al., 2019). However, there are still numerous challenges today (Glover and Manuck, 2018). Fortunately, recent studies suggest that high-throughput sequencing technologies may bring new hope to the prediction and therapy of PTB, for the close link between PTB and the results of whole-genome sequencing, exome sequencing, and transcriptome sequencing (Ngo et al., 2018; Strauss et al., 2018).

Circular RNA (circRNA) is a large class of non-coding RNAs formed naturally through the non-canonical splicing event called back-splicing (Costello et al., 2019). Since the first discovery by Sanger et al. in viroids in 1976, circRNA has long been considered as the by-products of splicing errors without biological significance (Patop et al., 2019). However, the latest studies have confirmed that circRNA not only widely expressed in human but also have multiple biological functions (Kristensen et al., 2019; Ottesen et al., 2019). For example, circRNA is conservative, tissue- and/or developmental stage-specific with a unique circular covalently bonded structure which means high stability, and therefore emerged as a promising molecular that could be an ideal biomarker of gastric cancer, heart failure, and other diseases (Devaux et al., 2017; Zhang et al., 2018). Also, circRNA is an essential competitive endogenous RNA (ceRNA) which could act as microRNA (miRNA) sponges through their abundance binding sites (miRNA response elements, MREs), and thus relieved the inhibition of miRNA on downstream target genes (circRNA–miRNA–mRNA mechanism) (Li et al., 2018).

At present, circRNA researches mainly focus on the field of cancer. CircRNA has been shown to play an essential regulatory role in the development and progression of various cancers such as hepatocellular carcinoma and lung cancer, via functioning sequester miRNAs and preventing its suppressive effect on target mRNAs (Cheng et al., 2018; Kristensen et al., 2018). Therefore, circRNA was regarded as a novel type of therapeutic target for cancers. For instance, in lung squamous cell carcinoma, circTP63 could promote proliferation and metastasis of cancer cells through competitively binds miR-873-3p to eliminate the inhibitory effect of miR-873-3p on cell cycle-related genes (Cheng et al., 2019). To date, only a few studies have demonstrated the biological role of circRNA in maternal–fetal health, which mainly focus on gestational diabetes and preeclampsia (Shafabakhsh et al., 2019). For example, circRNA_0001855 and circRNA_0004904 can not only involve in the pathology of preeclampsia by competitively binding miRNA but also predict preeclampsia even before 20 weeks of gestation (combined with PAPPA, total AUC = 0.940) (Jiang et al., 2018). In a word, circRNA, as a new class of molecules with biological activity, has been proved to be an important regulator and biomarker in many diseases including pregnancy-related complications. However, there have not been any published reports on the expression and functions of circRNAs in PTB.

Considering the advances of circRNA in the prediction and regulation of multiple diseases and the research gap in PTB, we analyzed the RNA-seq data and microarray data, to verify the hypothesize that circRNA may have the potential capacity to predict PTB as an effective biomarker and contribute to PTB via acting on downstream miRNA–mRNA. We discovered the circRNA disorders in PTB women for the first time. We proposed that the dysregulated circRNAs regulate, via the circRNA–miRNA–mRNA mechanism, various target genes involved in biological pathways, including the immune process, inflammation, and neural activity. Besides, three of these dysregulated circRNAs were confirmed to have considerable ability to diagnose PTB (AUC = 0.7138, 0.9589, 1.000). Therefore, our study provides new insight into the functions of circRNAs in PTB and lies a strong foundation for future research of PTB.

## Materials and Methods

### Microarray Expression Profile of circRNAs

The patients were recruited from the First Affiliated Hospital of Chongqing Medical University, and peripheral blood of them was obtained on admission. All pregnancies were single gestation without preeclampsia, fetal growth restriction, macrosomia, fetal distress, and any other pregnancy complications. The preterm group fulfilled the inclusion criteria of presenting the signs and symptoms of spontaneous preterm labor, such as regular uterine contraction. The term group was composed of pregnant women whose gestational age greater than 37 weeks regardless of whether they are in labor or not. After screening under these conditions, we finally obtained three preterm and three term samples. Blood samples (10 mL) were collected from the antecubital vein in EDTA (ethylenediaminetetraacetic acid)-containing tubes (Yuli, Jiangsu, China). All samples were centrifugated at 3000 rpm for 10 min at 4°C, and then the supernatant was transferred to another tube followed with repeatedly centrifugated. Total RNAs were extracted from samples using Trizol reagent (Invitrogen, Gaithersburg, MD, United States) and purified with the NucleoSpin^®^ RNA clean-up kit (MACHEREY-NAGEL, Germany) following the manufacturers’ instructions. Total RNAs were digested with RNase R (Epicentre, Illumina, Inc.) to remove linear RNAs, and was then reversely transcribed into cDNA. The consequent microarray analysis was completed by the Bioassay Laboratory of CapitalBio Corporation (Beijing, China).

Ethic approval was obtained from the Ethics Committee of the First Affiliated Hospital of Chongqing Medical University.

### Data Collection

All eligible RNA-seq data were downloaded from the Sequence Read Archive (SRA) database of the National Center for Biotechnology Information^1^. The selection criteria were as follow: (1) Total RNA sequencing with ribosomal and globin mRNA depleted. (2) The data were derived from whole peripheral blood of preterm and term pregnant women without any other complications. (3) The samples were taken when preterm labor symptoms appeared or their gestational age greater than 37 weeks. (4) Selected data should be raw data. Data from two paired-end RNA-seq projects (SRP107901 and SRP144363) were obtained after filtering. Overall, a total of 19 PTB and 16 full-term samples were included in our study.

### Data Processing

The downloaded SRA files were translated to paired-end FASTQ files using the fastq-dump command of SRA Toolkit (version 2.9.6-1). The RNA-seq reads were mapped to UCSC human reference genome (hg19)^2^ via the BWA-MEM algorithm. Then, CIRI2 (CircRNA Identifier 2) tool was used to predict and quantify circRNAs with default parameters, and gencode.v22.annotation.gtf was supplied as the annotation file (Gao et al., 2018). The overlap in circRNA identifications from all the datasets was visualized by TBtools software. The relative expression level of circRNAs was normalized by SRPBM (spliced reads per billion mapping) algorithm. Furthermore, since all samples were sequenced twice, the expression levels in the two results were averaged for subsequent research.

### Identification of Differentially Expressed circRNAs

Differentially expressed circRNAs between term and preterm groups were identified with the threshold value of fold-change >2 and *P*-value <0.05. This circRNA were annotated in detail through the circAtlas^3^, circBank^4^, and circBase^5^ database. To visualize these identified circRNAs, we used the TBtools software to generate the circular heatmap Upset plot. The volcano plot, cluster heatmap, and chromosome mapping diagram were generated by R software (version 3.6.2). The information about circRNAs expression in different tissues or organs was obtained from the circAtlas database (Wu et al., 2020).

### Construction of the ceRNA Network

The miRNA binding sites of circRNAs were predicted by circBank which integrates the result data from the miRanda^6^ and the TargetScan.^7^ The miRDB^8^ and the TargetScan (total context++ score ≤−0.6) were used to predict miRNA–targeted mRNAs. The ceRNA network was constructed using a combination of circRNA–miRNA pairs and miRNA–mRNA pairs. The Cytoscape 3.7.0 software was used to visualize the regulatory network.

### Functional Enrichment Analysis

To predict the function of the selected circRNAs, we performed the GO (Gene Ontology) analysis and the KEGG (Kyoto Encyclopedia of Gene and Genomes) pathway enrichment analysis via Metascape^9^ (Zhou et al., 2019). Terms with a *P*-value <0.01, a minimum count of 5, and an enrichment factor >1.5 were collected and grouped into clusters based on their membership similarities. Consequently, we used the ggplot2 package of R software (version 3.6.2) to draw histogram and bubble chart to show these results.

### Construction of the PPI Network and Module Analysis

The STRING (Search Tool for the Retrieval of Interacting Genes)^10^ is an online biological database for searching known protein interactions. The PPI (protein–protein interaction) network was constructed by STRING with the cut off criterion of confidence score ≥0.4, and the maximum number of interactors was set at “0” to reveal the potential relationship among those target genes. Moreover, the visualization of the PPI network was performed using Cytoscape software. The CytoHubba app was used to discover the hub genes.

### Statistical Analysis

Statistical analysis was performed using the SPSS (version 25.0, Chicago, IL, United States), GraphPadPrism (version 8.0, San Diego, CA, United States), and R (version 3.6.2) software. The circRNA expression data derived from microarray were normalized using the Quantile normalization method. The data generated by RNA-seq were normalized by the SRPBM algorithm. Mean and standard deviation (mean ± SD) of all data were calculated. The fold-changes were calculated by dividing the mean expression value of the preterm group by that of the term group. The student’s *t*-test was used to analyze the difference between groups and *P* < 0.05 (or FDR < 0.05) was considered statistically significant. The ROC (receiver operating characteristic) curves were constructed using the MedCalc software (version 10.0.7, Mariakerke, Belgium).

## Results

### CircRNA Expression Profiles in Preterm and Term Pregnant Women

**FIGURE 1:**
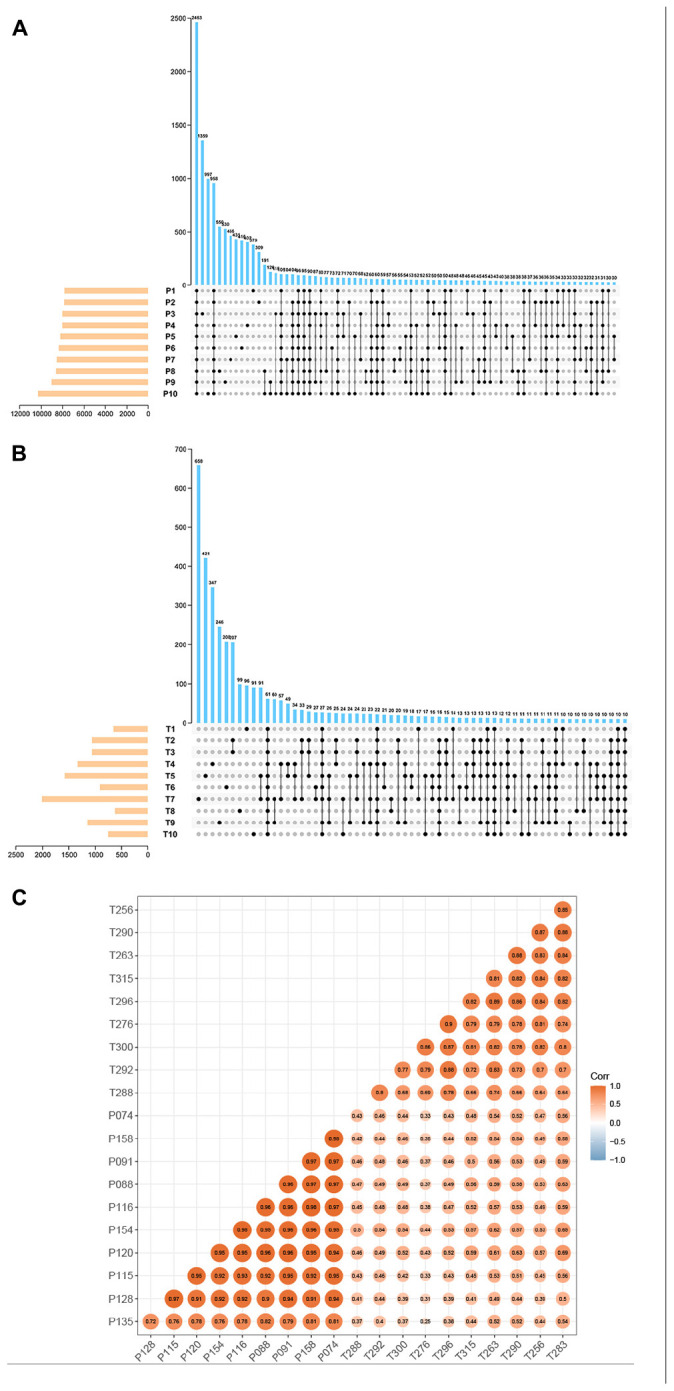
The basic characteristic of circRNA data. UpSet plot displaying the intersection of circRNAs identified in the different samples. **(A)** Preterm group (*n* = 10). **(B)** Term group (*n* = 10). **(C)** Heatmap of sample correlation. The color and size of each circle showed the strength of the correlation.

Total RNA-seq data of preterm and term cases were downloaded from the SRA database (accession number: SRP107901 and SRP144363). These two datasets contain the data of multiple types of RNA (mRNA, lncRNA, circRNA, etc.), in which circRNA information has never been identified. In total, 23704 circRNAs were identified in ten pairs of samples (preterm and term) by CIRI2, and the expression of them was normalized by the SRPBM algorithm to minimize potential batch effects. To ensure the reliability of the results, only circRNAs detected in more than five samples in each group were selected. The amounts of selected circRNAs reached 7948 in the preterm group and 673 in the term group (Figures 1A,B). Among them, 656 circRNAs detected in both groups were chosen for subsequent analysis. All shared circRNAs were annotated in detail via matching to the circAtlas, circBase, and circBank databases. A strong correlation regarding the circRNA expression of samples in each group and the discrepancy between the two groups are displayed by calculating the Pearson product-moment correlation coefficient amomng samples (Figure 1C).

### Dysregulated circRNAs

**FIGURE 2:**
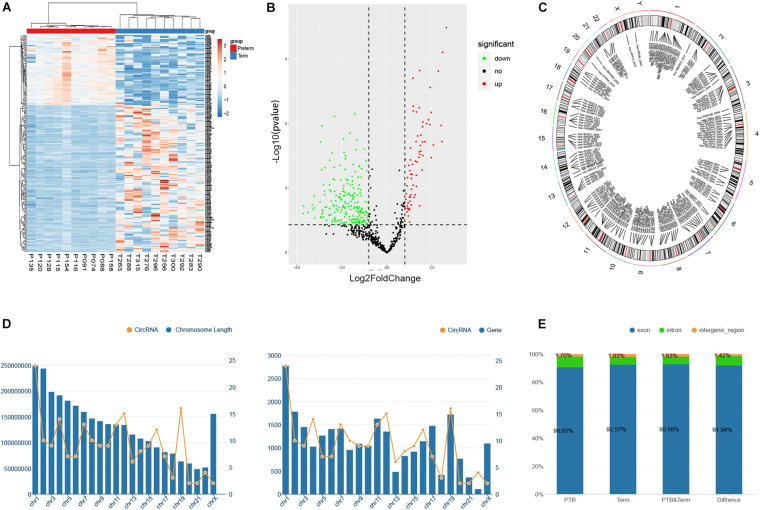
Identification of differentially expressed circRNAs between preterm and term samples. **(A)** Hierarchical clustering of selected circRNAs (*P* < 0.05). **(B)** Volcano showed differentially expressed circRNAs in two groups. **(C)** The chromosome distribution of significantly dysregulated circRNAs. **(D)** The relationship between the number of circRNAs distributed on chromosomes and the length and the number of genes of chromosomes, respectively. **(E)** The ratio of circRNAs originated from exonic, intronic, and intergenic regions.

In this study, the threshold that fold-change of greater than 2 and FDR (false discovery rate) smaller than 0.05 were used to detect the dysregulated circRNAs. As shown in volcano plots and cluster heatmap, a total of 211 eligible circRNAs were discovered, of which 68 circRNAs were upregulated and 143 circRNAs were downregulated in the preterm group (Figures 2A,B). The maximum fold-change of upregulated circRNA was 25.05, while the downregulated was 9.83.

To explore the origin of these differentially expressed circRNAs, the chromosomes and genomic regions were analyzed, of which the circRNAs were generated from. The results revealed that these circRNAs were widely distributed on all the human chromosomes, of course, except the Y chromosome (Figure 2C). Overall, the distribution of circRNA on chromosomes on chromosomes showed a gradually decreasing trend from chromosome 1 to chromosome X, which may be related to the length and gene number of each chromosome (Figure 2D). Genomic annotations revealed that exon-derived circRNAs account for 92.0% of all 211 dysregulated circRNAs, while 6.6% originated from the intron regions and 1.4% from intergenic regions. Simultaneously, we evaluated the proportion of circRNAs derived from exon, intron, and intergenic regions in the initially identified circRNAs of the preterm and term groups. However, there were no significant differences compared to that in the dysregulated circRNAs (Figure 2E).

### The circRNA–miRNA–mRNA Network

**FIGURE 3:**
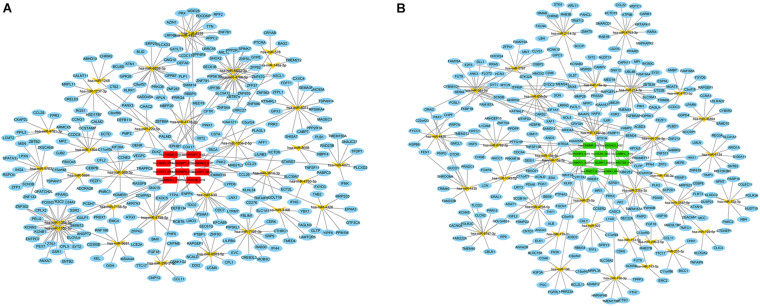
CeRNA (circRNA–miRNA–mRNA) regulatory network in PTB. **(A)** Red rectangle denotes upregulated circRNAs in the preterm group, **(B)** Green rectangle denotes downregulated circRNAs in the preterm group. Yellow angle denotes miRNAs, blue ellipse denotes mRNAs.

Since the primary function of circRNA is affecting gene expression levels by acting on miRNAs, we established the ceRNA network to further describe the regulatory mechanism of the dysregulated circRNAs. Firstly, the top 10 up- and down-regulated circRNAs in the preterm group were selected by fold change. The details of these circRNAs were displayed in Supplementary Table 1. Secondly, based on the identification of the MREs via circBank database, both up- and down-regulated circRNAs predicted 34 target miRNAs, respectively. Thirdly, by taking the intersection of the results from miRDB and TargetScan database, 314 and 308 downstream genes were predicted, respectively. Finally, the interactions between all the components were displayed in the ceRNA network, which was constructed by circRNA–miRNA and miRNA–mRNA pairs (Figure 3).

### Functional Enrichment Analyses of Target mRNAs

**FIGURE 4:**
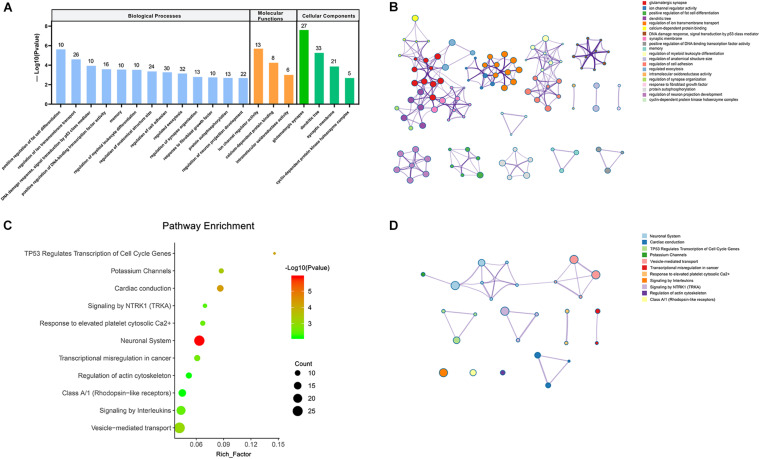
Visualizations of functional enrichment. **(A)** Top 20 enriched GO terms. **(B,D)** The network of enriched GO and KEGG terms colored by the cluster. **(C)** KEGG analysis for targeted genes (*P* < 0.01).

To get the in-depth biological function information of the dysregulated circRNAs, the KEGG pathway and GO analysis were performed to annotate the function of the target mRNAs within the above networks. The top 20 enrichment GO items were selected and ranked by *P*-value, and classified into three functional categories: biological processes (13 items), molecular functions (3 items), and cellular components (4 items). The genes were mostly enriched in several biological processes, including positive regulation of fat cell differentiation, regulation of ion transmembrane transport, regulation of myeloid leukocyte differentiation, regulation of synapse organization, regulation of neuron projection development, etc. These genes involved in the molecular functions such as ion channel regulator activity, calcium-dependent protein binding, intramolecular oxidoreductase activity, while participated in the cellular components, including glutamatergic synapse, dendritic tree, synaptic membrane, cyclin-dependent protein kinase holoenzyme complex (Figures 4A,B). Furthermore, the KEGG pathways in which these genes were significantly (*P* < 0.01) enriched included neuronal System, potassium Channels, vesicle-mediated transport, signaling by Interleukins, signaling by NTRK1 (TRKA), and regulation of actin cytoskeleton, etc. (Figures 4C,D).

### Establishment and Analysis of PPI Network

**FIGURE 5:**
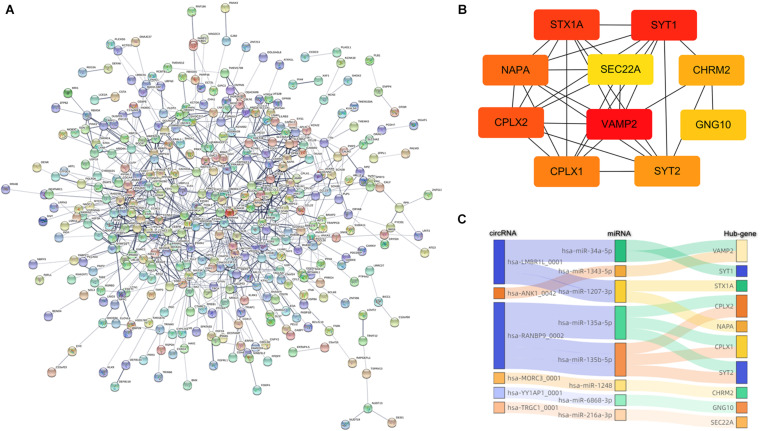
Construction of the PPI network. **(A)** Nodes and edges represent genes and interactions. The color of the connection line represented the type of interaction evidence. **(B)** Subnetwork of 10 hub genes that extracted from **(A)**, and the depth of the color indicates the level of the MCC score. **(C)** Sanky diagram to visualize the circRNA–miRNA–hubgene relationship.

As the biological process is usually carried out by a cluster of interrelated genes instead of a single one, a PPI network was constructed with a combined score ≥0.4 using the STRING database to analyze the interaction of these target genes and find hub genes. Genes that have no interaction with other genes were excluded based on evidence from previous experiments, co-expression analysis, and relevant databases. The final PPI network mapped 530 nodes and 833 edges with an average degree of 3.14 (Figure 5A).

Based on this visualization of interaction, we found the key subnetwork and hub genes of the circRNA regulatory system. Genes with the highest MCC (maximal clique centrality) score in the PPI network were considered as hub genes, which played an essential role in the network structure and might be the potential key genes of PTB. The top 10 hub genes were VAMP2, SYT1, STX1A, CPLX2, NAPA, CPLX1, SYT2, CHRM2, GNG10, and SEC22A. Among these genes, VAMP2 ranked first with a score of 1740 (Figure 5B). Combined with the previous ceRNA network, 6 circRNAs and 8 miRNAs that regulating these 10 hub genes were traced back, and circRNA-miRNA-hub gene regulatory pairs were shown in Figure 5C. These pairs were likely to be a key part of the PTB regulatory network, that is, potential therapeutic targets.

### Validation of PTB-Related circRNAs

To confirm the effectiveness of the candidate circRNAs as biomarkers for the prediction of PTB, validation experiments were performed by two steps.

**FIGURE 6:**
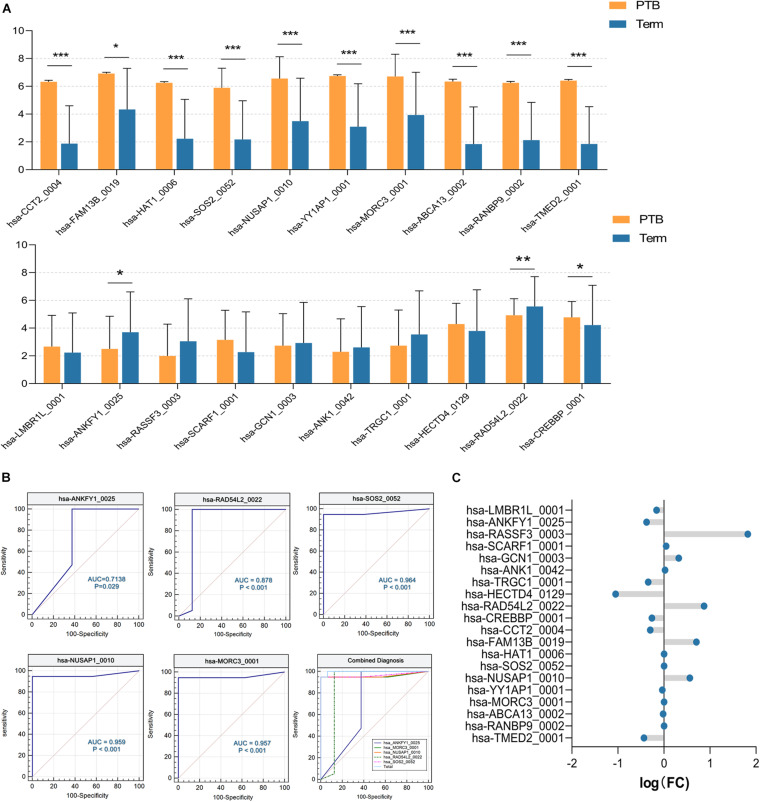
The validation of candidate circRNAs. **(A)** The expression level of circRNAs. *P < 0.05; **P < 0.01; ***P < 0.001. **(B)** ROC curve analysis of 5 selected circRNAs, and their integrated diagnostic efficiency (*P* < 0.05) (PTB, *n* = 19; Term, *n* = 16). **(C)** The fold change of circRNAs, which was calculated according to microarray data (PTB, *n* = 3; Term, *n* = 3).

Firstly, we expanded the sample size to 35 (19 preterm and 16 term samples) using the remaining data from SRP107901 and SRP144363 datasets. As shown in Figure 6A, significant differences between preterm and term group were indicated in 12 out of 20 aforementioned circRNAs, and the up-regulated or down-regulated trend remained consistent with the previous results (*P* < 0.05). On this basis, the ROC curve was selected to analyze the diagnostic efficiency of candidate circRNAs for PTB. There were 7 circRNAs that had AUC (area under curve) value greater than 0.98: hsa-CCT2_0004 (AUC = 0.9836), hsa-HAT1_0006 (AUC = 0.9901), hsa-FAM13B_0019 (AUC = 1), hsa-YY1AP1_0001 (AUC = 1), hsa-ABCA13_0002 (AUC = 1), hsa-RANBP9_0002 (AUC = 1) and hsa-TMED2_0001 (AUC = 1). And the AUC of hsa-ANKFY1_0025, hsa-RAD54L2_0022, hsa-SOS2_0052, hsa-NUSAP1_0010, and has-MORC3_001 were lower than that of the above 7 circRNAs, reaching 0.7138,0.8783, 0.9638, 0.9589 and 0.9572, respectively. But interestingly, using logistic regression analysis, we found that the combined diagnosis of the above five circRNAs worked well (AUC = 0.9970), which is far more effective than any single one of them (Figure 6B). All of these 12 circRNAs above might have the potentials to be biomarkers for PTB.

Furthermore, circRNA microarray assays targeting candidate circRNAs were performed. The fold-changes of candidate circRNAs were shown in Figure 6C. There were slight but significant differences of hsa-ANKFY1_0025, hsa-FAM13B_0019, hsa-NUSAP1_0010 between two groups, which appears to match the decrease/increase trend of RNA-seq results above (Supplementary Table 2). It suggested that the abnormal expression of these three circRNAs in the preterm group was stable, which might be used as an effective biomarker for PTB.

## Discussion

Due to the severe harm of PTB to neonates and the limitations of the current prediction and treatment, it is necessary to research the pathogenesis of PTB and search for its biomarkers (Muglia and Katz, 2010). There has been an explosion of interest and study on circRNA in various diseases since 2013 when it was first reported to be related to the occurrence of Alzheimer’s disease (AD), due to its stable nature and its ability to regulate a variety of biological pathways through the ceRNA mechanism (Lukiw, 2013; Zhong et al., 2018). Like AD, PTB is closely related to immune inflammation, so it is worthy of thinking whether circRNAs can trigger PTB via the same regulatory mechanism as in AD. Our data, for the first time, revealed the circRNA expression profile of the peripheral blood from preterm and term pregnant women. Besides, we constructed the circRNA-miRNA-mRNA network to explore the regulatory function of dysregulated circRNAs in PTB and performed ROC analysis to assess the predictive value of circRNAs for PTB.

By analyzing the RNA-seq data downloaded from the SRA database, we observed noticeable disorders of circRNA expression in the whole peripheral blood of preterm women compare to term women. Generally, the top 10 up- and down-regulated circRNAs filtered by fold-change (range from 5.24 to 25.05) were more likely to have a stable association with PTB. Eleven of these 20 circRNAs have never been reported before, and we speculated that their dysregulation might be specific for PTB. The remaining 9 circRNAs have been demonstrated to be correlated with breast cancer (hsa-SCARF1_0001), osteoarthritis (hsa-GCN1_0003), osteosarcoma (hsa-RAD54L2_002), virus infection (hsa-CREBBP_0001), ovarian cancer (hsa-FAM13B_0019), hepatocellular carcinoma (hsa-NUSAP1_0010), endometrial cancer (hsa-YY1AP1_0001), hypopharyngeal squamous cell carcinoma (hsa-MORC3_0001), and gestational diabetes mellitus (hsa-RANBP9_0002) (Fu et al., 2018; Kun-Peng et al., 2018; Hu et al., 2019; Li et al., 2019; Lou et al., 2019; Wang et al., 2019, 2020; Guo et al., 2020; Tran et al., 2020). Interestingly, we found that all these diseases shared a common pathological ground of inflammation and immune activation with PTB, which happens to be the main way that the selected circRNAs regulate these diseases (Cappelletti et al., 2016). For example, hsa-GCN1_0003 promotes the secretion of TNF-α in synovial tissue through the ceRNA mechanism, and TNF-α has been confirmed to be elevated in PTB (Helmo et al., 2018). Several other immune/inflammation-related factors (such as IFN-γ, IL-10, etc.) mentioned in the above reports, have been proved to be involved in the pathological cascade reactions of PTB (Pandey et al., 2017; Frascoli et al., 2018). So, we are wondering whether these circRNAs participate in PTB via the inflammatory/immune pathway. Additionally, another piece of evidence comes from the parental genes, of which the circRNAs were derived from. Since circRNAs can activate the transcription of their parental genes, the function of these genes could indirectly reflect the biological impact of circRNAs to some extent (Fischer and Leung, 2017). Among all parental genes of selected circRNAs, CREBBP (the gene of hsa-CREBBP_0001) has been noted to affect the inflammatory signal (NF-κB pathway) of the myometrium, and closely related to labor onset and PTB (Zeng et al., 2008; Wagner et al., 2017). Thus, hsa-CREBBP_0001 was suggested to be associated with PTB by affecting the inflammatory pathway. Based on these hypotheses and evidence, we concluded that the biological function of these dysregulated circRNAs in PTB seems to be related to immune/inflammation. To understand their roles in PTB, the circRNA–miRNA–mRNA network and subsequent function analysis were needed.

In the present study, we predicted the downstream miRNA–mRNA of the maladjusted circRNAs and established a ceRNA network containing 20 circRNAs, 68 miRNAs, and 622 genes to analysis their potential regulation mode. Notably, the targeted mRNAs seem mainly related to immune cell differentiation, interleukin signaling, and nervous system. The first two of these three items were not only consistent with the above circRNA functions, but also widely reported in the researches of PTB. It is generally considered as an essential feature of PTB that maternal immune cells (such as neutrophils, T cells) are activated in advance, differentiated, and recruited rapidly to the uterus, maternal–fetal interface, and other tissues and thus triggering an inflammatory cascade reaction (Erlebacher, 2013; Gomez-Lopez et al., 2014). For example, before the symptoms of PTB appeared, CD4^+^T cells in maternal circulation and uterus were differentiated into phenotypes such as Th1 and Th17, breaking the immune balance which is required to maintain pregnancy, and releasing inflammatory factors such as IL-17 and TNFα (Ito et al., 2010). Even CD4^+^T cells in fetal cord blood proliferate and differentiate into the IFNγ-producing phenotype, thereby promoting the inflammatory effects associated with PTB (Frascoli et al., 2018; Halkias et al., 2019). The immune cell differentiation leads to the activation of downstream IL-1, IL-6, IL-33, and other interleukin signaling pathways (Cappelletti et al., 2016). Among them, IL-1 and IL-6 have always been regarded as the most significant contributor to the occurrence of PTB. Their expression levels and receptors activities have increased significantly, activating NF-κB, Jak/Stat, and other inflammatory pathways to trigger PTB (Nadeau-Vallée et al., 2015; Pandey et al., 2017). In our circRNA-miRNA-mRNA network, IL-6R (IL-6 receptor) was directly regulated by hsa-LMBR1L_0001 and has-miR-1207-3p. Currently, the specific causes of PTB-related immune cell differentiation and interleukin-signaling enhancement are not clear. We propose a new idea that abnormal circRNAs in preterm pregnant women can affect a variety of genes (including IL-6R, IFNγ) by binding to miRNAs, thereby regulating the immune/inflammatory pathway and ultimately promoting PTB. Besides, our results also suggest that circRNAs may regulate the nervous system functions through the ceRNA mechanism, such as “regulation of synapse organization” and “regulation of neuron projection development.” Considering that the most significant symptom of premature infants is neurological system damage, we speculate that the disordered circRNA in maternal blood may reflect or regulate fetal neural development to a certain extent. Still, further research is needed.

By searching for hub genes, we found that circRNA–miRNA–mRNA regulatory pairs played a crucial role in the whole network, which were regulated by 6 circRNAs including hsa-MORC3_0001 and hsa-LMBR1L_0001. One of the most striking pair was hsa-MORC3_0001–hsa-miR-1248–CHRM2. Hsa-miR-1248 was found significantly decreased in peripheral blood leukocytes of preterm women, and involved in the positive regulation of interleukin−2 signaling by binding to gene SASH3 and CD83 (Paquette et al., 2019). Interleukin−2, one of the proinflammatory factors mainly secreted by activated CD4^+^ Th1 cells, was reported to be elevated and stimulated the contraction of myometrium in PTB (Sykes et al., 2012). Besides, the activated CD4^+^ Th1 cells also produced proinflammatory IL-1β and contributed to the activation of the IL-1β pathway, which is one of the classical signaling pathways that regulate PTB development (Nadeau-Vallée et al., 2016). Coincidentally, *in vitro* experiments, CHRM2, the downstream gene of hsa-miR-1248 in our results, was demonstrated to be engaged in the PTB-related inflammatory process of decidual cells responding to IL-1β signaling (Ibrahim et al., 2016). CHRM2, encoding the Cholinergic Receptor Muscarinic 2, controlled the contractility of smooth muscles in the lung, colon, and other tissues (Patten et al., 2015). Thus, there was a possibility that hsa-MORC3_0001–hsa-miR-1248–CHRM2 pair might plays a vital role in both IL-1β and IL-2 inflammatory process produced by CD4^+^ Th1 cells, consequently leading to myometrial contraction, and ultimately giving rise to PTB. Besides hsa-miR-1248, hsa-miR-135a-5p, which regulated by hsa-RANBP9_0002 has been found increased in the placenta tissues of PTB (Fallen et al., 2018). Moreover, hsa-LMBR1L_0001, as mentioned above, regulated the expression of IL-6R by binding to has-miR-1207-3p. The remaining three pairs have been rarely reported in PTB, and were mainly reported to influence the growth, migration, and invasion of tumor cells by regulating genes related to the adaptive and innate immune response in various cancers including pancreatic cancer (Yonemori et al., 2017; Felix et al., 2019). Given the importance of these six regulatory pairs on our ceRNA network and their role in regulating immune/inflammation processes, they may have the potential to be the therapeutic targets for PTB.

As previously mentioned, the risk of PTB might be increased by multiple factors such as metabolic disorders, progesterone withdrawal, intestinal flora imbalance, and psychological stress, besides immune/inflammation (Romero et al., 2014). And recent studies have shown that circRNA disorders indeed interact with these risk factors. For example, it has been found that a cluster of circRNA in the endometrium can be regulated by progesterone in goat, thereby affecting the endometrial receptivity to the embryo (Song et al., 2019). Also, a specific circRNA (circ_0002861) can regulate progesterone secretion in the ovarian of mice (Jia et al., 2018). So, is the progesterone withdrawal associated with PTB-related circRNA dysregulation? There has been no previous evidence. Similarly, metabolic disorders (e.g., obesity) are contributors to PTB, and circRNAs have been shown to play a primary role in regulating energy metabolism in cancer. Therefore, we hypothesized that circRNA may also mediate the promotion effect of other risk factors on PTB, which needs to be confirmed by further studies.

Comparing to be a regulator, circRNA may have the unique advantage to be a biomarker. At present, two of the most commonly used biomarkers for PTB prediction were fFN and placental alpha-macroglobulin-1 (PAMG-1; Suff et al., 2019). These two tests were usually performed in the third trimester of gestation (25-34 weeks gestation) and had notable high NPV (97.9%, 98.3%) but low PPV (7.9%, 35.3%) for the prediction of PTB within 7 days (Wing et al., 2017). However, misdiagnosis can lead to unnecessary treatment or a higher risk of PTB, and late prediction of PTB often makes clinical interventions extremely difficult, so a new efficient and reliable alternative test should be developed. As a non-invasive detection method, circRNA may have more potential. By analyzing RNA-seq data, we identified 12 circRNAs as promising biomarkers. Seven of them showed an extremely high diagnostic value for PTB (AUC > 0.98). The AUC of the other five circRNAs ranged from 0.71 to 0.98, while their combined diagnostic performed higher efficiency (AUC = 0.997, 95% CI = 0.894–1.000, sensitivity = 94.7%, specificity = 100%). Furthermore, we used microarray data to identify the stability of these biomarkers, and finally, three optimal circRNAs were selected: hsa-ANKFY1_0025, hsa-NUSAP1_0010, and hsa-FAM13B_0019(AUC = 0.7138, 0.9589, 1.000). Currently, there are no reports of them as biomarkers, but we found some interesting features of them from the circAtlas database. All of them displayed evident tissue-specific expression. In normal cases, Hsa-NUSAP1_0010 and hsa-ANKFY1_0025 were enriched in nervous system tissue (brain and spinal cord), which was coincidence with the PTB-related potential neural change discussed previously. The last 4–6 weeks of pregnancy is the critical period for fetal neurodevelopment, and the brain weight of the neonate who was born at 34 weeks is only 65% of that of the term neonate (Scher et al., 2011). Moreover, hsa-FAM13B_0019 was highly expressed in the myometrium, which is the main effector of PTB. Maybe this is the reason why these three circRNAs could be selected as suitable biomarkers of PTB. Therefore, we wondered if the biological homeostasis of the uterus and the fetal nervous system had been disrupted before the onset of preterm labor, and the tissues releasing these three circRNAs that generally accumulated in these tissues. That is, circRNA has the potential to be a reliable biomarker to predict intrinsic changes before the appearance of clinical symptoms of preterm labor, thus providing sufficient time for the clinical intervention. Besides, our data were derived from pregnancies between 28 and 35 weeks of gestation (as same as the fFN and PAMG-1 tests), but it is not clear when circRNAs began to change. Thus, it is indeed a matter of concern whether circRNAs can predict PTB at an earlier time point.

There are still some limitations to our study. First, the sample size was small, so more samples are needed to confirm the predictive value of circRNAs for PTB. Second, the actual function of circRNA may be affected by the complex biological factors in pregnant women, for example, the level of oxidative stress may affect circRNA expression (Li et al., 2018), and changes in gut microbes may affect the regulation of metabolic function by circRNA(Zhu et al., 2020), and these factors may vary widely among individuals. Therefore, we will carry out miRNA/mRNA-seq, molecular experiments, and animal experiments, to further exclude potential confounding factors and analyze the actual effect of circRNA on preterm delivery. Third, our results have not indicated the optimal detection time point for circRNA to predict PTB, and we will explore this issue through prospective cohort study in the next step.

In conclusion, through systematic bioinformatic analysis and validation experiments, we proposed for the first time that circRNA is likely to become of tremendous research value in the prediction and mechanism of PTB, and pioneered a new field of PTB research. We found that 211 abnormally expressed cricRNAs existed in the peripheral blood of preterm women. Among them, the top 20 circRNAs with the highest fold-changes competitively bond to 68 miRNAs, including hsa-miR-1248, thereby regulating 622 downstream genes (such as CHRM2, etc.) related to inflammation, immunity and neural activity, thus ultimately involving in the process of PTB. Six pairs, especially hsa-MORC3_0001–hsa-miR-1248–CHRM2, are the key parts of the entire regulatory network and might be the breakthrough point in researching the mechanism of PTB. Furthermore, hsa-ANKFY1_0025, hsa-FAM13B_0019, and hsa-NUSAP1_0010 (AUC = 0.7138, 0.9589, 1.000) might have the potential to be novel predictive indicators of PTB. This study offered a new strategy for PTB prediction and proposed a novel type of mechanism theory of PTB that is worth further study to confirm.

## Data Availability Statement

The original contributions presented in the study are included in the article/Supplementary Material, further inquiries can be directed to the corresponding author.

## Ethics Statement

The studies involving human participants were reviewed and approved by Medical Research Ethics Committee of The First Affiliated Hospital of Chongqing Medical University. The patients/participants provided their written informed consent to participate in this study.

## Author Contributions

HQ and NY designed the experiments and supervised the study. YR and DH collected and analyzed the data. YR wrote the manuscript. YZ, JY, and HZ revised the manuscript. All authors contributed to the article and approved the submitted version.

## Conflict of Interest

The authors declare that the research was conducted in the absence of any commercial or financial relationships that could be construed as a potential conflict of interest.

## Footnotes

1 https://www.ncbi.nlm.nih.gov/sra/

2 http://www.genome.ucsc.edu

3 http://circatlas.biols.ac.cn/

4 http://www.circbank.cn/

5 http://www.circbase.org/

6 http://www.microrna.org/microrna/home.do

7 http://www.targetscan.org/vert_72/

8 http://mirdb.org/

9 http://metascape.org/gp/index.html

10 http://string.embl.de/
