# Supplementary material for: Identification and Characterization of Circular RNA as a Novel Regulator and Biomarker in Preterm Birth
Source: Front Bioeng Biotechnol. 2020 Dec 2;8:566984. doi: 10.3389/fbioe.2020.566984 (PMC7775733; doi:10.3389/fbioe.2020.566984)
Supplement: Supplementary file 1 [file Table_1.DOCX]

**Supplementary Table 1.** The details of top 10 up- and down-regulated circRNA in the preterm group

|  | **CircAtlas_ID** | **CircBase_ID** | **CircBank_ID** | **Chromosome** | **Start** | **End** | **Fold change** | **FDR** |
| --- | --- | --- | --- | --- | --- | --- | --- | --- |
| **Down-regulated** | hsa-LMBR1L_0001 | hsa_circ_0004602 | hsa_circLMBR1L_015 | chr12 | 49491349 | 49491888 | 25.05 | 0.04412 |
|  | hsa-ANKFY1_0025 | hsa_circ_0106897 | hsa_circANKFY1_026 | chr17 | 4092677 | 4096155 | 24.53 | 0.02689 |
|  | hsa-RASSF3_0003 | - | - | chr12 | 65042317 | 65043332 | 20.69 | 0.04078 |
|  | hsa-SCARF1_0001 | hsa_circ_0000732 | hsa_circSCARF1_001 | chr17 | 1540003 | 1540356 | 17.57 | 0.02517 |
|  | hsa-GCN1_0003 | hsa_circ_0000448 | hsa_circGCN1L1_117 | chr12 | 120592774 | 120593523 | 16.98 | 0.03297 |
|  | hsa-ANK1_0042 | hsa_circ_0084084 | hsa_circANK1_005 | chr8 | 41561559 | 41571772 | 16.20 | 0.01240 |
|  | hsa-TRGC1_0001 | - | - | chr7 | 38282029 | 38299831 | 16.18 | 0.02962 |
|  | hsa-HECTD4_0129 | hsa_circ_0028381 | hsa_circC12orf51_096 | chr12 | 112645678 | 112657293 | 13.04 | 0.01977 |
|  | hsa-RAD54L2_0022 | hsa_circ_0001306 | hsa_circRAD54L2_001 | chr3 | 51575514 | 51586079 | 12.80 | 0.00016 |
|  | hsa-CREBBP_0001 | hsa_circ_0007637 | hsa_circCREBBP_007 | chr16 | 3900298 | 3901010 | 12.20 | 0.00147 |
| **Up-regulated** | hsa-CCT2_0004 | hsa_circ_0000418 | hsa_circCCT2_007 | chr12 | 69983265 | 69985939 | 5.24 | 4.27176E-05 |
|  | hsa-FAM13B_0019 | hsa_circ_0001535 | hsa_circFAM13B_024 | chr5 | 137320946 | 137324004 | 5.31 | 2.28963E-06 |
|  | hsa-HAT1_0006 | hsa_circ_0008032 | hsa_circHAT1_002 | chr2 | 172782047 | 172809519 | 5.52 | 1.79378E-05 |
|  | hsa-SOS2_0052 | hsa_circ_0007695 | hsa_circSOS2_047 | chr14 | 50616726 | 50616948 | 5.56 | 0.00057 |
|  | hsa-NUSAP1_0010 | hsa_circ_0002124 | hsa_circNUSAP1_014 | chr15 | 41667910 | 41669502 | 5.99 | 0.00021 |
|  | hsa-YY1AP1_0001 | hsa_circ_0014606 | hsa_circYY1AP1_008 | chr1 | 155646339 | 155649303 | 7.26 | 5.88914E-07 |
|  | hsa-MORC3_0001 | hsa_circ_0001189 | hsa_circMORC3_020 | chr21 | 37711077 | 37717005 | 8.08 | 4.38412E-05 |
|  | hsa-ABCA13_0002 | hsa_circ_0001707 | hsa_circABCA13_004 | chr7 | 48541722 | 48542148 | 8.34 | 0.00016 |
|  | hsa-RANBP9_0002 | hsa_circ_0001578 | hsa_circRANBP9_023 | chr6 | 13639795 | 13644961 | 8.54 | 1.60247E-07 |
|  | hsa-TMED2_0001 | hsa_circ_0000458 | hsa_circTMED2_002 | chr12 | 124071294 | 124074996 | 9.83 | 2.26104E-08 |
