# Supplementary material for: Identification and Characterization of Circular RNA as a Novel Regulator and Biomarker in Preterm Birth
Source: Front Bioeng Biotechnol. 2020 Dec 2;8:566984. doi: 10.3389/fbioe.2020.566984 (PMC7775733; doi:10.3389/fbioe.2020.566984)
Supplement: Supplementary file 2 [file Table_2.DOCX]

**Supplementary Table 2.** The detailed information of circRNAs selected by multiple methods.

|  | **RNA-seq screening_FC** | **RNA-seq validation_FC** | **ROC** | | | **Microarray_FC** |
| --- | --- | --- | --- | --- | --- | --- |
|  |  |  | **AUC** | **Sensitivity** | **Specificity** |  |
| **hsa-ANKFY1_0025** | 0.0408 | 0.0426 | 0.7138 | 100.0% | 62.5% | 0.7694 |
| **hsa-NUSAP1_0010** | 5.9852 | 9.7380 | 0.9589 | 94.7% | 100.0% | 1.4755 |
| **hsa-FAM13B_0019** | 5.3108 | 6.0930 | 1.0000 | 100.0% | 100.0% | 1.6303 |
